# Dual/Multi-Modal Image-Guided Diagnosis and Therapy Based on Luminogens with Aggregation-Induced Emission

**DOI:** 10.3390/molecules29020371

**Published:** 2024-01-11

**Authors:** Linlin Zhu, Wenbo Wu

**Affiliations:** Department of Chemistry, Institute of Molecular Aggregation Science, Tianjin University, Tianjin 300072, China; linlinzhu_julie@tju.edu.cn

**Keywords:** aggregation-induced emission, dual/multi-modal imaging, diagnosis, theragnostic, phototherapy

## Abstract

The combination of multiple imaging methods has made an indelible contribution to the diagnosis, surgical navigation, treatment, and prognostic evaluation of various diseases. Due to the unique advantages of luminogens with aggregation-induced emission (AIE), their progress has been significant in the field of organic fluorescent contrast agents. Herein, this manuscript summarizes the recent advancements in AIE molecules as contrast agents for optical image-based dual/multi-modal imaging. We particularly focus on the exceptional properties of each material and the corresponding application in the diagnosis and treatment of diseases.

## 1. Introduction

Nowadays, imaging technology is increasingly indispensable for the diagnosis and treatment of various diseases [1,2,3], which undoubtedly presents great opportunities for the development of contrast agents. Organic contrast agents enjoy a high reputation because of their excellent properties, such as good biocompatibility and multifunctionality [4,5,6]. However, the aggregation-caused fluorescence quenching (ACQ) effect of most conventional organic fluorophores largely limits their further applications [7,8,9]. On the contrary, contrast agents with aggregation-induced emission (AIE) [10,11,12] have brought the dawn of imaging technology due to their ease of accumulation in vivo [13], and facilitation of signal conduction and capture in the aggregated state. Additionally, AIE luminogens (AIEgens) also exhibit several other advantages, including high brightness, robust photobleaching resistance, and the absence of random blinking [14,15]. Structural modifications can further regulate the molecular motion of AIEgens, thereby controlling both radiative and non-radiative decay, which is beneficial for balancing the fluorescence and other types of signals for dual/multi-modal imaging, or even balancing the image and therapy functions for theranostics [10,16,17,18,19,20,21,22,23]. Therefore, the development of high-performance AIE contrast agents is becoming one of the focuses in this field.

According to previous reports [24,25,26,27], powerful AIE contrast agents usually possess two main characteristics. Firstly, they are capable of dual/multi-modal imaging to cater to various imaging requirements. Secondly, in practical applications, they integrate multiple functions, including imaging and therapy, together into one single agent. Traditional imaging techniques, such as computed tomography imaging (CTI) [28], magnetic resonance imaging (MRI) [29], ultrasound imaging (USI) [30], positron emission tomography (PET) imaging [31], etc., are commonly used for diagnostics. These imaging methods offer certain advantages, yet they share a common and undeniable shortcoming: the possibility of errors when imaging subtle changes at the molecular level. Recently, some other imaging techniques, such as fluorescence imaging (FLI) [32], dark-field microscopy (DFM) imaging [33], photoacoustic imaging (PAI) [34], Raman imaging (RI) [35], magnetic particle imaging (MPI) [36], and photothermal imaging (PTI) [37], have also emerged for disease diagnosis. Among them, AIEgen-based FLI allows for clear, sensitive, and high spatiotemporal resolution imaging at the molecular level [38,39,40]. However, FLI has a critical limitation in penetration depth, which hinders the diagnosis of deep-seated lesions. Therefore, by combining AIEgen-based FLI with the aforementioned imaging techniques, it is possible to overcome the limitations of single imaging modality and leverage the complementary structural and functional information provided by each imaging approach, resulting in enhanced diagnostic performance.

**Figure 1:**
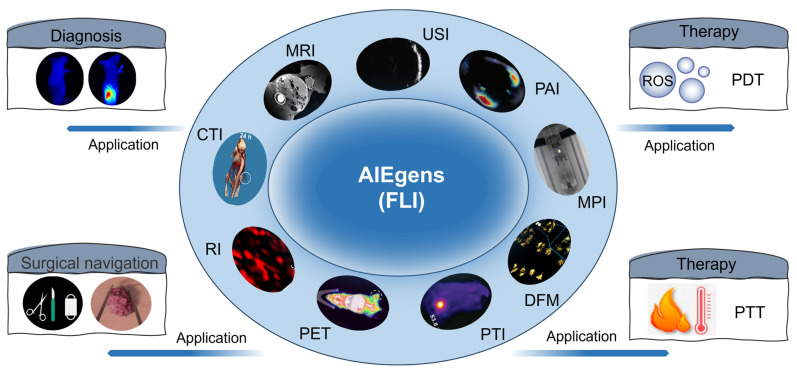
Dual/multi-modal image-guided diagnosis and therapy based on AIEgens with FLI as the basic function.

In the past few years, several reviews have been published to discuss the progress of dual/multi-modal imaging [41,42,43,44], due to its advantages in the diagnosis of diseases. However, none of them focused on the AIE type contrast, although they show several advantages, as discussed before. Therefore, in this review, we would like to summarize the latest progress of AIEgens in dual/multi-modal image-guided diagnosis and therapy (Figure 1). The relationships among structures, properties, and applications in the diagnosis and treatment of diseases are specially focused upon during our discussion.

## 2. AIEgen-Based Dual-Modal Imaging

In this part, the dual-modal imaging through FLI with traditional imaging techniques, including CTI, MRI, and PET, are first introduced. As compared to these traditional imaging methods alone, the joint usage of FLI techniques can offer enhanced resolution and sensitivity for molecular-level imaging, thereby obtaining more precise imaging information. Subsequently, the combination of FLI with the emerging techniques of PAI or PTI was also summarized. In comparison with FLI, PAI provides deeper penetration and more accurate delineation of tumor edges, and, therefore, is attractive in biomedical applications [45,46,47,48]. In addition, benefiting from AIEgens’ inherent ability as a special mechanism to subtly regulate the balance between radiative and non-radiative decay, they would show special advantages in FLI/PTI dual-modal imaging with simultaneous photothermal therapy (PTT). 

### 2.1. Dual-Modal Imaging of FLI and CTI

**Figure 2:**
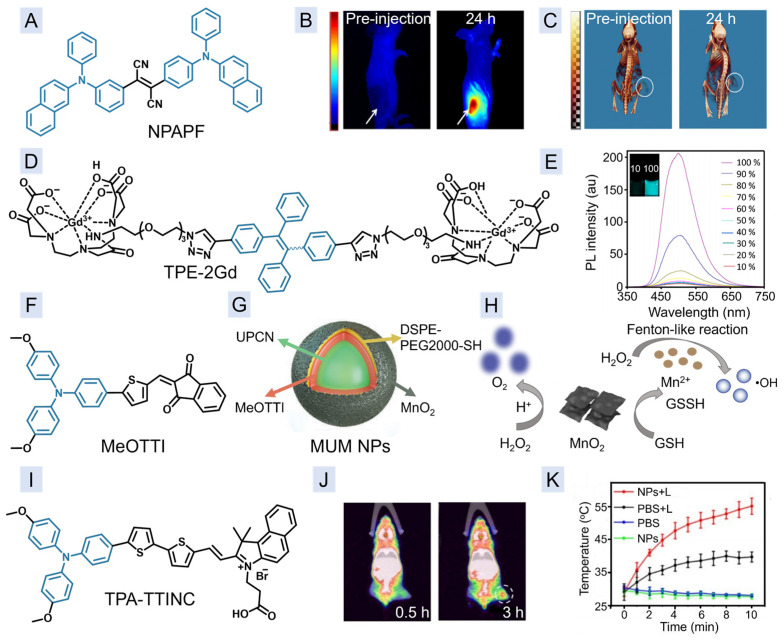
(**A**) Chemical structure of NPAPF. (**B**,**C**) The FLI (**B**) and CTI (**C**) images of CT26 tumor bearing mice before and after injection of M-NPAPF-Au NPs (the tumor sites are marked with white arrows or circles). (**D**) Chemical structure of TPE-2Gd. (**E**) The FL spectra of TPE-2Gd in solutions with different fractions of water (THF/water). The inset picture is the fluorescence contrast photos taken under 365 nm UV irradiation at water fractions of 10% and 100%. (**F**) Chemical structure of MeOTTI. (**G**) Composition of MUM NPs. (**H**) Schematic illustration of the role of MnO_2_ in MUM NPs. (**I**) Chemical structure of TPA-TTINC. (**J**) PET images of tumor-bearing mice after injection of TPA-TTINC NPs for different times. (**K**) Temperature changes in tumors in mice after different treatments. (**B**,**C**) Reprinted with permission from [47]. Copyright 2015, Elsevier. (**E**) Reprinted with permission from [52]. Copyright 2014, American Chemical Society. (**G**,**H**) Reproduced with permission from [53]. Copyright 2021, John Wiley and Sons. (**J**,**K**) Reproduced with permission from [54]. Copyright 2023, John Wiley and Sons.

Benefiting from high spatial resolution and infinite penetration depth, CTI technology is widely recognized by clinical medicine [49,50]. In 2015, Liang et al. reported a unique nanoprobe, M-NPAPF-Au, which was prepared by simultaneous encapsulating AIEgen NPAPF (Figure 2A) and computed tomography (CT) contrast agent gold nanoparticles (NPs) into DSPE-PEG2000 micelles [51]. After being encapsulated into NPs, NPAPF can keep its AIE feature, endowing M-NPAPF-Au NPs with the dual-modal imaging ability of FLI and CTI (Figure 2B,C).

### 2.2. Dual-Modal Imaging of FLI and MRI

MRI is another highly recognized clinical imaging method which can utilize nuclear magnetic resonance to provide nuclear relaxation times T1 and T2, and which offers superior advantages over CTI in acquiring detailed organ and soft tissue images, including nerves, blood vessels, and muscles [29,55]. The corresponding AIEgen-based contrast agents are usually designed by the complexation AIEgens with metal ions [56,57], or by coating them with manganese dioxide [53] or superparamagnetic iron oxide [58].

As early as 2014, Tang’s group reported the first example of AIEgen-based dual-modal imaging of FLI and MRI [52]. As shown in Figure 2D, TPE-2Gd is composed with one hydrophobic tetraphenylethene (TPE) and two hydrophilic gadolinium diethylenetriaminepentaacetic acid moieties. Such an amphiphilic structure makes TPE-2Gd self-assemble into nano micelles in aqueous solution with a critical micelle concentration of 70 μM. The introduction of a TPE group makes the whole molecule exhibit typical AIE properties (Figure 2E) and high brightness in aqueous solution for FLI. In addition, as an MRI contrast agent, TPE-2Gd has a longitudinal relaxation of 3.36 ± 0.10 s^−1^ per mM of Gd^3+^, similar to that of the commercial reagent magnevist (3.70 ± 0.02 s^−1^ per mM of Gd^3+^). Importantly, thanks to its nano-sized structure, the circulating life of TPE-2Gd in living mice is prolonged to 1 h, much longer than that of magnevist (10 min), offering longer imaging time. 

The therapeutic function can also be attached onto the contrast agents, and MUM NPs (Figure 2F,G) is one of typical examples [53]. The AIE photosensitizer (PS) MeOTTI was precipitated with rare earth-doped upconversion NPs (UCNPs) by using DSPE-PEG2000-SH as the polymer matrix to form MU NPs, which would undergo the redox reaction between sulfhydryl units and KMnO_4_ to form the final MnO_2_-shelled MUM NPs. The introduction of MeOTTI endowed MUM NPs with aggregation-induced near-infrared (NIR) emission for FLI, and type-I reactive oxygen species (ROS) generation capability for photodynamic therapy (PDT). The MnO_2_ shell also plays a significant role in MUM NPs. Firstly, MnO_2_ could degrade H_2_O_2_ to O_2_ by leveraging its catalase-like ability, alleviating intracellular hypoxia. Secondly, MnO_2_ can react with overexpressed GSH in the tumor site to generate Mn^2+^ for T_1_-weighted MRI. Thirdly, the generated Mn^2+^ can further convert H_2_O_2_ to •OH through a Fenton-like reaction with the help of CO_2_/HCO_3_^−^ for killing cancer cells. Thus, the highly efficient FLI-MRI-guided PDT could be achieved. 

### 2.3. Dual-Modal Imaging of FLI and PET Imaging

PET imaging was developed relatively late. In terms of disease diagnosis, PET imaging is more inclined to depict molecular metabolism. The primary strength of PET imaging over CTI and MRI is its ability to diagnose early-stage conditions, particularly when the morphological structure of the lesion site remains unchanged.

TPA-TTINC in Figure 2I is a photothermal agent with aggregation-induced emission in second near-infrared window (NIR-II) range [54]. After being chelated with the radionuclide ^68^Ga, the corresponding TPA-TTINC NPs would yield ^68^GA-TPA-TTINC NPs for dual-modal imaging of FLI in NIR-II range and PET imaging with a high tumor/muscle ratio of 4.8 (Figure 2J) [54]. Subsequently, under the guidance of dual-modal imaging, the PTT of breast tumor could also be realized (Figure 2K). 

### 2.4. Dual-Modal Imaging of FLI and PAI

PAI is an emerging non-invasive imaging method that converts optical signals into ultrasound wave signals, providing high spatial resolution and good penetration at centimeter levels [59,60,61]. In comparation with traditional CTI, MRI, and PET imaging technologies, PAI possess advantages, such as low cost, non-radiation, simple operation, and so on [62,63,64,65,66,67,68,69,70,71,72,73,74,75,76,77]. When PAI is combined with FLI, it can compensate for FLI’s lack of penetration depth [63,64,65,66,67,68]. For AIEgens, their intramolecular rotation and/or vibration for non-radiative decay make themselves naturally excellent contrast agents for PAI [69], which is very helpful in designing dual-modal imaging agents of FLI and PAI.

**Figure 3:**
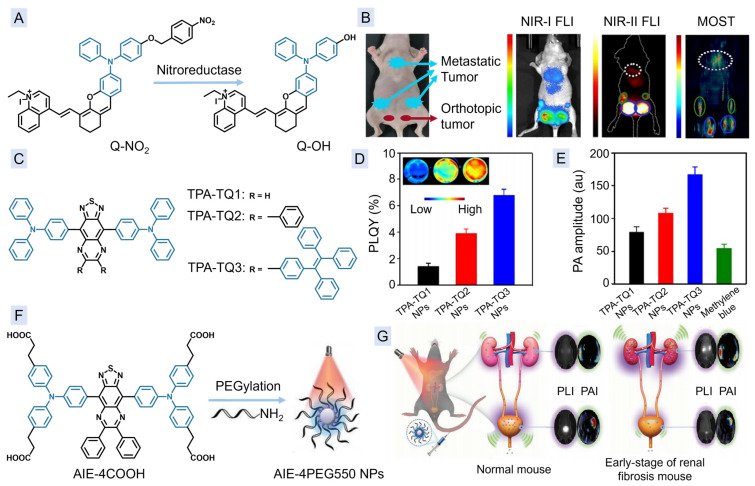
(**A**) Chemical structure of Q-NO_2_, as well as its reaction with nitroreductase. (**B**) The FLI in the first NIR (NIR-I) window, NIR-II FLI, and MSOT images of the mice after injection of Q-NO_2_ for 30 min. (**C**) Chemical structures of TPA-TQ1, TPA-TQ2, and TPA-TQ3. (**D**) The photoluminescence QYs of TPA-TQ1 NPs, TPA-TQ2 NPs, and TPA-TQ3 NPs (inset are the corresponding brightness images of NPs). (**E**) PA amplitudes of TPA-TQ1 NPs, TPA-TQ2 NPs, TPA-TQ3 NPs, and methylene blue. (**F**) The chemical structure of AIE-4COOH and the preparation of AIE-4PEG550 NPs. (**G**) Schematic illustration of FLI/PAI dual-modal detection of mouse nephropathy by AIE-4PEG550 NPs. (**B**) Reproduced with permission from [63]. Copyright 2019, John Wiley and Sons. (**D**,**E**) Reproduced with permission from [64]. Copyright 2021, John Wiley and Sons. (**G**) Reproduced with permission from [65]. Copyright 2022, John Wiley and Sons.

Multispectral optoacoustic tomography (MSOT) is one type of functional PAI technique, which captures ultrasonic signals generated by various light absorbers upon irradiation with multiple wavelengths. Therefore, MSOT allows for easy acquisition of three-dimensional (3D) PAI of targeted tissues. In 2020, Wu and co-workers developed an activatable dual-modal imaging probe of MSOT and FLI for monitoring breast cancer metastasis [63]. Comparing with contrast agents with constant fluorescence and photoacoustic signals, activatable PAI/FLI agents have greater advantages in the specific detection and diagnosis of diseases. As shown in Figure 3A, the nitrobenzyloxydiphenylamino group was introduced to the strong electronic donor–acceptor system to yield a probe, Q-NO_2_, without photoacoustic and fluorescent signals. Upon specific reaction with nitroreductase, one of the cancer markers, Q-OH is released, leading to the restoration of corresponding photoacoustic and fluorescence signals. Accordingly, the probe enables the dual-modal imaging of both orthotopic and metastatic breast tumors (Figure 3B).

The fluorescence and PA signals arise from radiative and non-radiative decays, respectively, and are typically in competition with each other, which means that it is usually difficult to enhance both signals simultaneously. In 2021, Ding et al. attempted to improve the thermal-to-acoustic conversion for achieving relatively high PA signals through low non-radiative transitions, resulting in the enhancement of both fluorescence and PA signals [64]. They prepared a series of thiadiazoloquinoxaline (TQ)-based AIEgens, namely TPA-TQ1, TPA-TQ2, and TPA-TQ3, with different side groups of H, phenyl, and TPE (Figure 3C). With the introduction of more rotator groups, the AIE activity becomes more pronounced, leading to the highest fluorescence quantum yield (QY) of TPA-TQ3 NPs (Figure 3D). Additionally, the rotator structures can enhance the intramolecular motions, and subsequently improve the thermal-to-acoustic conversion, so that TPA-TQ3 NPs can also demonstrate the highest photoacoustic (PA) signals (Figure 3E) with the lowest photothermal conversion efficiency of only 18.7%. Subsequent in vivo experiment revealed that TPA-TQ3 NPs are helpful in providing unambiguous guidance for tumor surgery through dual-modal imaging of FLI and PAI. Shortly after that, Tang, Wang, and co-workers realized the hydrophilization of such an agent for chronic kidney disease (CKD) detection [65]. The PEGylation of the AIEgen through amidation reaction could yield AIE-4PEG550 (Figure 3F), which could self-assemble into NPs with an average diameter of only 26 nm, making them suitable for renal clearance. Specially, the renal clearance efficiency of AIE-4PEG550 NPs was up to 93.1 ± 1.7% 24 h after the injection. As a consequence, the longitudinal fibrosis staging of renal was achieved by the dual-modal imaging of NIR-II FLI and PAI (Figure 3G).

### 2.5. Dual-Modal Imaging of FLI and PTI

PTI is another imaging method that also utilizes non-radiative transitions, which can provide images of photo-induced temperature differences with the assistance of photothermal agents (PTAs). Therefore, before PTT, PTI alone or PTI-based dual-modal imaging of PTI were usually employed to provide guidance for treatment [22,78,79,80,81,82,83].

**Figure 4:**
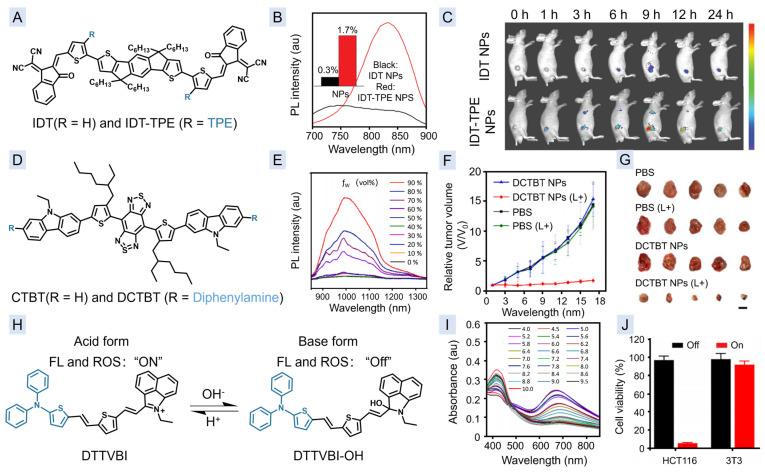
(**A**) Chemical structures of IDT and IDT-TPE. (**B**) Fluorescence spectra and absolute QYs of IDT NPs and IDT-TPE NPs. (**C**) FLI of mice after injection with IDT NPs or IDT-TPE NPs for different times. (**D**) Chemical structures of CTBT and DCTBT. (**E**) The fluorescence spectra of DCTBT in THF solutions with different fraction of water. (**F**,**G**) Tumor relative volume changes (**F**) and tumor images (**G**) in different treatment groups. (**H**) Structure and properties change in DTTVBI NPs in different acid-base environments. (**I**) Absorption spectrum change in DTTVBI NPs in different pH. (**J**) Cell viabilities of DTTVBI NP-treated HCT 116 cancer cells or 3T3 normal cells before and after laser irradiation. (**B**,**C**) Reproduced with permission from [22]. Copyright 2021, John Wiley and Sons. (**E**–**G**) Reprinted with permission from [78]. Copyright 2022, with permission from Elsevier. (**I**,**J**) Reprinted with permission from [79]. Copyright 2023, American Chemical Society.

In 2021, a new AIEgen IDT-TPE [22] was developed by Tang, Wang, and co-workers through simply linking two TPE groups as the rotators onto the side chains of an NIR fluorophore IDT (Figure 4A) [22]. In comparison with IDT NPs, IDT-TPE NPs exhibits red-shifted absorption and emission centered at around 690 and 835 nm, respectively, and a higher QY of 1.7% (Figure 4B), which are suitable for PTI, and FLI, respectively. In addition, upon 660 nm excitation, IDT-TPE NPs could also produce ROS for PDT. Therefore, by using IDT-TPE NPs as the agents, combination tumor phototherapy of PDT and PTT could be realized under the guidance of dual-modal imaging of FLI (Figure 4C) and PTI. Shortly after that, the same research group developed another agent, namely DCTBT (Figure 4D), by the same strategy, but using diphenylamine group as the rotators [78]. In comparison with IDT-TPE NPs, the excitation wavelength and emission of DCTBT NPs was red-shifted to 808 nm, and the NIR-II range (Figure 4E), which is beneficial for improving the penetration depth. As a consequence, by using DCTBT NPs as the agent, the PANC-1 pancreatic tumors were treated with high efficacy (Figure 4F,G) through NIR-II FLI/PTI-guided simultaneous PDT and PTT in a mouse model. 

Recently, the activable NIR-II FLI/PTI theranostic agent was also developed [79], which allows for more precise treatment with decreased side effects. DTTVBI exhibits distinct chemical structures (Figure 4H) with different intramolecular charge transfer (Figure 4I), making it show NIR-II emission and type-I photosensitization only in acid environments. Therefore, DTTVBI NPs could specifically kill cancer cells upon excitation (Figure 4J), due to the tumor acidic microenvironment [80]. In the patient-derived tumor xenograft (PDX) model, significant tumor growth inhibition without any side effects was observed under the guidance of dual-modal imaging of NIR-II FLI and PTI.

## 3. AIEgen-Based Multi-Modal Imaging

### 3.1. Multi-Modal Imaging of FLI, CTI, and DFM Imaging

Dark-field microscopy (DFM) imaging is an imaging technique that utilizes reflected and diffracted light to observe objects [84,85,86,87]. While regular microscopy can detect details as small as 0.45 μm, DFM can capture extremely small objects ranging from 0.2 to 0.004 μm. The large scattering cross-section of DFM helps to avoid the interference caused by ensemble averaging effects in optical scattering imaging and single nanoparticle analysis [88]. Due to the irradiation method used in DFM, it only visualizes the outline or movement of the object.

**Figure 5:**
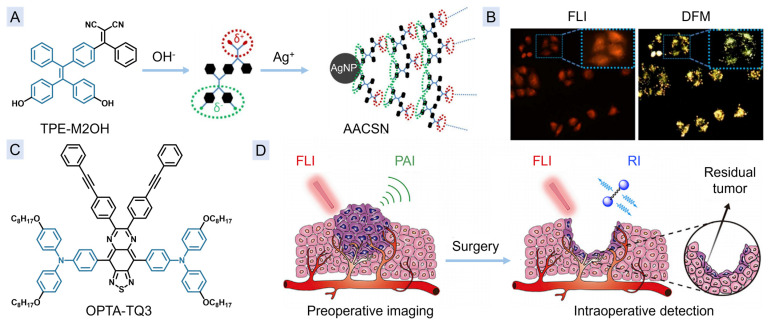
(**A**) The preparation of AACSN. (**B**) The fluorescence and DFM images of HeLa cells after incubation with AACSN (inset images representative the amplified selected area). (**C**) Chemical structure of OPTA-TQ3. (**D**) Schematic illustration of surgical treatment guided by FLI/PAI/RI multi-modal imaging based on OPTA-TQ3 NPs. (**A**,**B**) Reproduced with permission from [89]. Copyright 2018, American Chemical Society. (**D**) Reprinted with permission from [20]. Copyright 2019, with permission from Elsevier.

In 2018, a core–shell NP AACSN was reported to realize the multi-modal imaging of FLI, CTI, and DFM [89]. The preparation of AACSNs involved a redox reaction and subsequent self-assembly process, using a redox AIEgen (TPE-M2OH) containing a phenol group and Ag^+^ as precursors. The introduction of self-assembled AIEgen-based plasma to the noble metals NP can avoid the severe loss of fluorescence signals usually caused in simple direct combination of fluorophores and noble metals through Förster resonance energy transfer or electronic transfer (Figure 5B), and this type of localized surface plasmonic resonance (LSPR) of noble metals can be used for DFM imaging with a high signal-to-noise ratio (Figure 5B) [85,86,87]. Furthermore, the Ag NP-based CTI is also able to provide high spatial resolution and deep tissue penetration for multimodal imaging. The multi-modal imaging incorporated the advantages of high signal-to-noise ratio of FLI and DFM, as well as the deep penetration depth of CTI, thereby showing significant potential in cancer diagnostics.

### 3.2. Multi-Modal Imaging of FLI, PAI, and RI

Surgical treatment of complex tumors necessitates the use of robust imaging materials for accurate mapping of tumor edges. In addition to the complementary nature of FLI and PAI in terms of sensitivity and penetration, RI can produce high contrast images by utilizing its cell-silence region at 1800–2800 cm^−1^ [90,91,92]. In 2019, OPTA-TQ3 (Figure 5C) was designed by using phenyl-alkyne-phenyl, which could give a strong Raman signal at around 2200 cm^−1^, to modify the side chains of an NIR emissive AIEgens, into which FLI, PAI, and RI were integrated together [20]. In a mouse model, OPTA-TQ3 NPs has been successfully used for preoperative imaging of the tumor region under the guidance of FLI-PAI and the detection of intraoperative tiny residual tumor under the guidance of FLI-RI (Figure 5D). Guidance by this multi-modal imaging, all the tumor range could be excised without residues.

### 3.3. Multi-Modal Imaging of FLI, PAI, and PTI

**Figure 6:**
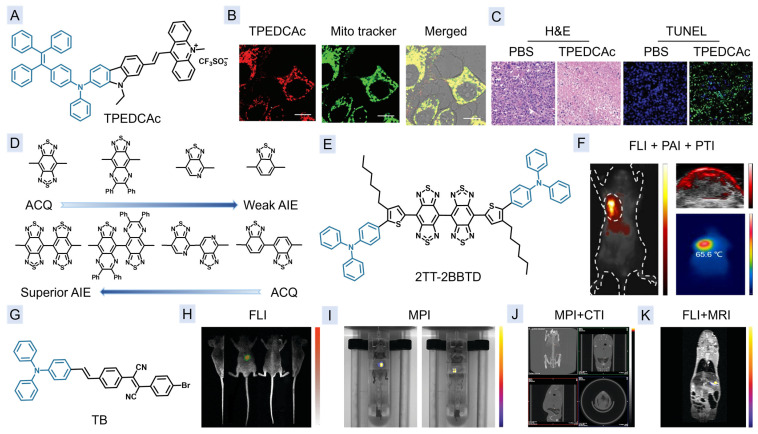
(**A**) Chemical structure of TPEDCAc. (**B**) The FLI images of MCF-7 cells after continuous incubation with TPEDCAc and Mito Tracker Green. (**C**) The therapeutic effect of TPEDCAc upon 660 nm laser excitation tested by H&E and TUNEL staining analyses. (**D**) Chemical structures and AIE characteristics of different acceptors. (**E**) Chemical structure of 2TT-2BBTD. (**F**) NIR-II FLI, PAI image and PTI images of 4T1 tumor-bearing mice after injection of 2TT-2BBTD NPs. (**G**) Chemical structure of TB. (**H**–**K**) The FLI images (**H**), 2D MPI images (**I**), 3D MPI-CT fusion images (**J**), and MPI-MRI fusion images (**K**) after in situ injection with TSP NP-labeled HuH-7 cells. (**B**,**C**) Reproduced with permission from [93]. Copyright 2022, John Wiley and Sons. (**F**) Reprinted with permission from [94]. Copyright 2023, American Chemical Society. (**H**–**K**) Reproduced with permission from [108]. Copyright 2019, Royal Society of Chemistry.

Due to potential issues with surgical treatment, such as recurrence, researchers are exploring the possibility of combining the contrast agent with a therapeutic agent. Thus far, a range of multi-modal image-guided therapy cases have been reported, with most possessing longer emission wavelengths and AIE properties, which are particularly advantageous for achieving balance in FLI, PAI, and PTI [93,94,95,96,97,98,99,100,101,102,103,104,105,106,107]. The work of Tang et al. in 2022 is very representative [93]. They prepared a series of agents with the same donor, but different acceptors of the pyridinium, quinolinium, and acridinium unit, among which, TPEDCAc (Figure 6A) with an acridinium acceptor demonstrated the best performance, since the enhanced electron-withdrawing ability and size of the acridinium acceptor could redly shift the absorption and emission and helped to create more room for skeleton distortion for the AIE property, respectively. Upon 660 nm laser excitation, TPEDCAc demonstrated both the highest ROS generation and photothermal conversion efficiencies for PDT and PTI/PTT, respectively. Furthermore, only its emission wavelength could extend to the NIR-II range, indicating the deeper penetration depths in FLI. In addition, TPEDCAc could also target the mitochondria of the cancer cells, which is helpful in phototherapy (Figure 6B). In an MCF-7 tumor-bearing mice model, TPEDCAc is proven to be effective in inhibiting tumor metastasis through the multi-modal imaging-guided combination therapy of PDT and PTT (Figure 6C).

In order to study how the number of conjugated acceptors affect the AIE activity, a series of fluorophores with the same donor were prepared [94]. As shown in Figure 6D, the fluorophores with a single acceptor (1A) or two acceptors (2A) in donor–acceptor–donor (D-A-D) structures exhibited completely opposite trends in terms of AIE activity. In the 1A system, the AIE property increases as the electron withdrawing ability of the acceptor weakens, while the 2A system displays an opposite trend of ACQ to AIE. Accordingly, 2TT-2BBTD (Figure 6E) with the strongest electron acceptor in 2A system possessed the longest absorption wavelength with the highest molar extinction coefficient for photothermal conversion, and aggregation-induced NIR-II emission for FLI. Therefore, 2TT-2BBTD NPs were well-suited for multi-modal imaging of FLI, PAI, and PTI (Figure 6F). Furthermore, the ROS generation and photothermal conversion ability of 2TT-2BBTD NPs have been successfully utilized in eliminating breast cancer in living mice.

### 3.4. Multi-Modal Imaging of FLI, MPI, CTI, and MRI

Magnetic particle imaging (MPI) is an emerging imaging method that works by measuring the position and concentration of superparamagnetic iron oxide (SPIO) as a magnetic contrast agent [109]. The superparamagnetic properties of SPIO, combined with its compatibility with the human body and ease of metabolism, make MPI a safe 3D imaging method without the use of radiation [108]. Furthermore, MPI also possesses several other advantages, including quantitation, zero background interference, and deep penetration [110,111]. With the assistance of MPI, the intricate anatomical features and the spatial location of the lesion in the body can be delineated more clearly. In 2019, Tian et al. [108] prepared TSP NPs by encapsulating AIEgen TB (Figure 6G) and Fe_3_O_4_ into a polystyrene-polyethylene glycol (PS-PEG) matrix. The excellent superparamagnetism and relaxivity, as well as high PLQY of 14.6%, make TSP NPs outstanding multimodal contrast agents for multi-modal imaging of FLI, MPI, CTI, and MRI, allowing for the monitoring of orthotopic liver tumors with deep penetration and high spatial resolution.

## 4. Conclusions

In this work, the recent progress in AIEgen-based dual/multi-modal image-guided diagnosis and therapy was summarized in detail. By using dual/multi-modal imaging, it is possible to transcend the constraints of individual imaging modalities and capitalize on the complementary structural and functional information offered by each imaging technique, thereby improving diagnostic accuracy. As contrast agents, the AIEgens demonstrated vast potential, due to their high brightness in NIR or even NIR-II ranges under physiological environments, and due to the ease of balancing radiative and non-radiative decays. In addition, similar to other organic/polymeric materials, the AIEgens are easy to functionalize, which is beneficial for designing dual/multi-modal imaging agents and even theranostic agents.

Despite these advantages, the AIEgen-based dual/multi-modal imaging agents still have a certain distance to go before clinical translation. The first thing to consider is their safety, which requires a further detailed evaluation of their systemic toxicity, biodistribution, pharmacokinetics, metabolism, etc. However, it seems that only limited attention has been focused on this point, compared to enhancing imaging performance, at present. Another concern is that the multi-function nature of the agents has been only proven in the xenograft model of mice, and more deep and detailed studies are still necessary to evaluate how would they really perform in humanoid models before starting clinical conversion. Anyhow, we hope this review can encourage more and more interest in this area, for realizing its clinical application as soon as possible.

## Data Availability

No new data were created or analyzed in this study. Data sharing is not applicable to this article.
